# Atomistic Polymer Modeling: Recent Advances and Challenges in Building and Parametrization Workflows

**DOI:** 10.1021/acs.macromol.5c01166

**Published:** 2025-10-28

**Authors:** Hannah N. Turney, Micaela Matta

**Affiliations:** Department of Chemistry, 4616 King’s College London Strand Campus (East Wing), London WC2R 2LS, United Kingdom

## Abstract

Synthetic polymers are a broad and versatile class of soft materials covering a large chemical space. “Computational microscopy” approaches such as atomistic molecular dynamics (MD) simulations are an effective tool to validate and rationalize experimental data for structure–property characterization. The predictive quality of MD simulations and the properties derived from them are primarily driven by the accuracy and relevance of the force field used to represent the system. While biomolecular simulation (nucleic acids, proteins) workflows benefit from dedicated toolkits and domain-specific force fields, the modeling of synthetic polymers has not progressed to the same extent. This perspective will discuss recent efforts to improve system building and parametrization workflows for synthetic polymers, and the unique challenges differentiating them from biopolymers. We will outline shortcomings in established workflows, review best practices for FAIR polymer simulations, and highlight new tools/workflows leveraging cheminformatics, direct chemical perception, and neural networks.

## Graphical abstract

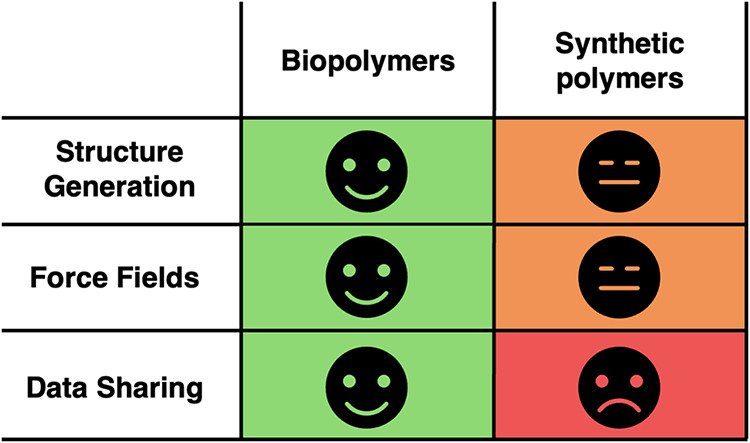

## Introduction

The biopolymer simulation community has made remarkable strides in part due to widespread adoption of FAIR (Findable, Accessible, Interoperable, and Reusable) simulation practices.^1^ Initiatives such as MDDB^2^ and MDverse,^3^ developed to make MD data sets accessible and increase transparency in their generation, have drawn broad support from researchers worldwide. The hallmark structural stability of protein molecules and functional nucleic acids allows for accurate static 3D representations and the prediction of single conformations. This property has been instrumental in the success of machine learning approaches such as AlphaFold as it provides a well-defined structural target that simplifies the calculation of loss functions.

These efforts have accelerated progress across the field, contributing to major milestones such as whole-cell simulations,^4^ drug optimization,^5,6^ and the collaborative characterization of the coronavirus spike protein.^7,8^ In contrast, the simulation of synthetic polymers has only begun to realize its potential. Despite their structural and functional diversity and range of applications,^9–12^ synthetic polymers remain comparatively underexplored in terms of large-scale, coordinated FAIR-compliant simulation efforts.

Although MD simulations have been used to study polymers for drug delivery/formulation,^13^ biodegradable plastics,^14,15^ coatings,^16,17^ energy production/storage,^18,19^ and high-performance composites,^20^ their full potential remains unexplored. MD simulations are often performed *a posteriori* rather than as part of an established material design pipeline. This is in contrast to biopolymer simulations which are performed routinely as an integral part of protein/drug design and ligand binding.^21–24^

Studies in the literature have used MD simulations to predict properties relevant to polymer physics such as miscibility,^25^ glass transition temperature,^20^ and mechanical properties.^26^ Experimental structural analysis techniques such as X-ray scattering are widely used to probe molecular complexity in polymers across a range of length scales, including nm scales for some techniques.^27,28^ For instance, small-angle X-ray scattering (SAXS) is particularly useful for examining the microstructure and chain flexibility of semicrystalline polymers.^29^ SAXS and MD are often used in tandem, with SAXS serving both as a complementary tool to MD and a means of experimentally validating polymer model development and parametrization.^30^

Despite significant advances in the capabilities of MD simulations, there is still a lack of standardized workflows for synthetic polymer modeling, compared to those available for biopolymers. Standardizing molecular dynamics workflows for synthetic polymers presents unique challenges compared to biopolymer systems. Key difficulties include: (i) the accurate *in silico* representation of high molecular weight macromolecules through single line notation or molecular files, (ii) the extraction and, where necessary, the modification of force field parameters at the monomer level, and (iii) the systematic mapping of these parameters onto polymer chains. Additionally, generating realistic, reproducible starting structures for amorphous materials without using long equilibration protocols is a major barrier.^31^

The discrepancy between parametrization guidelines for biopolymers and synthetic polymers (Figure 1) forms the basis of this perspective. It highlights the need for more robust and standardized methodologies to accurately capture the physical and chemical behaviors of a range of synthetic polymer chemistries with atomistic force fields, achieving comparable accuracy, reproducibility, and efficiency as domain-specific biopolymer force fields. Such advancements are critical for achieving reliable insights into their structure–property relationships and optimizing their performance across diverse applications.

**Figure 1:**
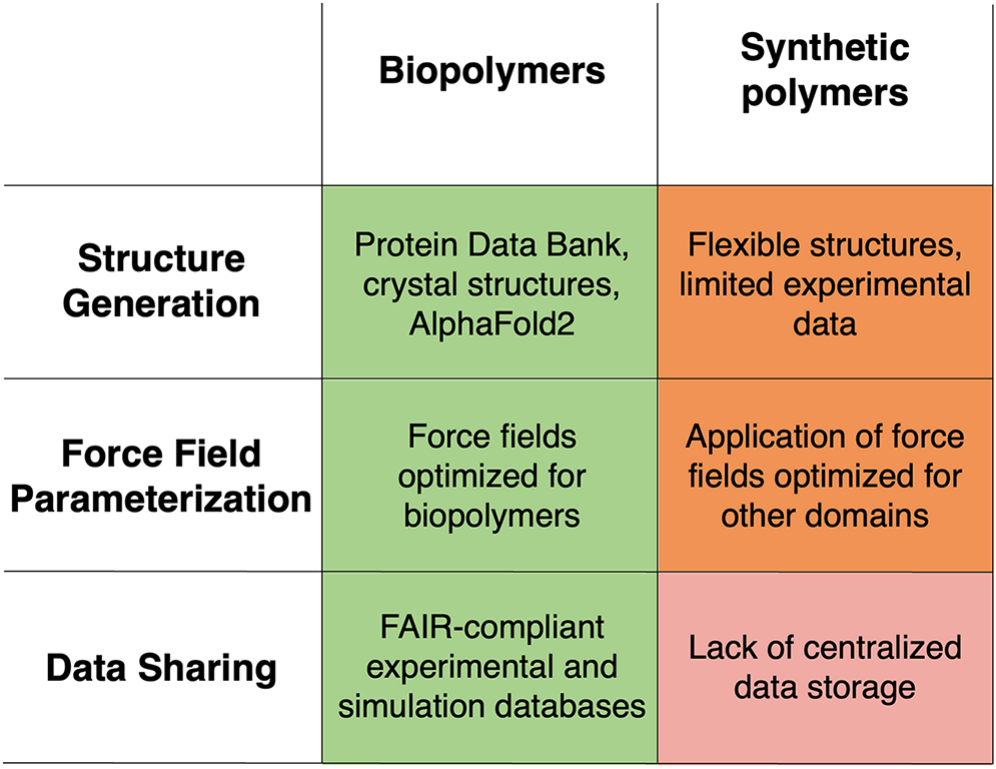
Current barriers in the building and parametrization of synthetic polymer systems for Molecular Dynamics.

This perspective will discuss existing challenges and new approaches to the setup of atomistic MD simulations of synthetic polymers, including tools and workflows for polymer parametrization, system building,^32^ and force fields. We outline the shortcomings in established tools and highlight solutions to achieve FAIR^33^ (Findable, Accessible, Interoperable, and Reusable) and reproducible^34^ polymer simulations (Figure 2). We will discuss the applications of coarse-graining in the preparation of atomistic models, but will not include a detailed account of advances in polymer coarse-grained simulations, a topic extensively reviewed in the literature.^35–39^ We do not attempt to capture definitive best practices for polymer modeling, done previously by Gartner and Jayaraman.^32^ Annealing protocols, machine learning interatomic potentials for potential energy surface estimation,^40,41^ simulation engines, and trajectory analysis will also be omitted.^32^

**Figure 2:**
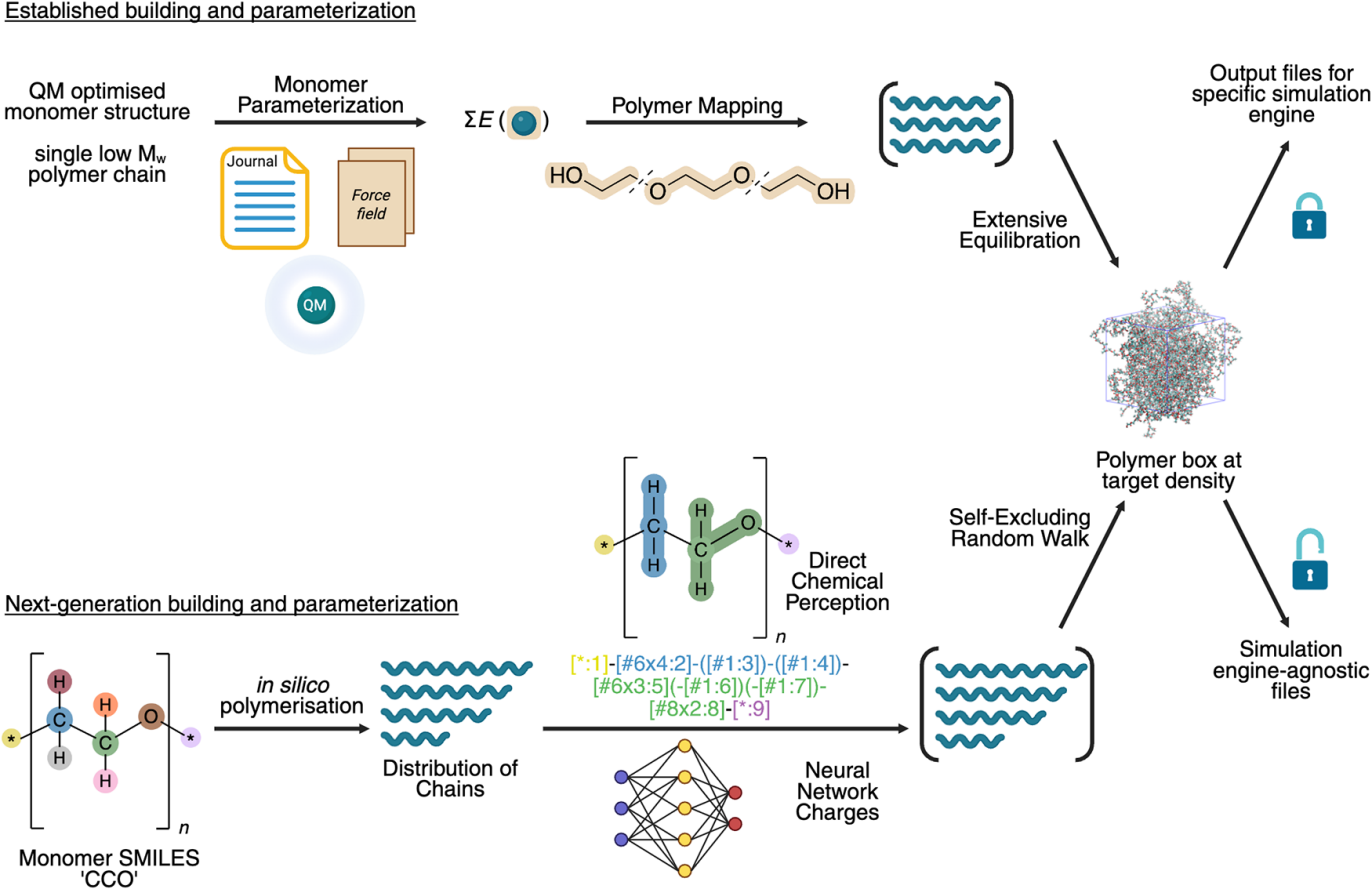
Established practices, and a proposed new process for preparing a synthetic polymer molecular dynamics system. Created in BioRender. Turney, H. (2025) https://BioRender.com/far2r3r.

## Challenges in Polymer Building, Atom Typing, and Parametrization Workflows

### Building Polymer Structures

When preparing a synthetic polymer model, challenges arise at key stages, including the generation of its 2D structure and connectivity, 3D conformation, and building an initial configuration for MD simulation. These steps play a role in determining the accuracy and reliability of downstream parametrization and simulation impacting the model’s applicability.^31,46^ Inaccurate initial structures can lead to unrealistic polymer configurations that bias simulations and make it difficult to explore the full conformational space of polymers, hindering the prediction of mechanical, thermal, and transport properties.^42–44^ More advanced conformer sampling techniques and enhanced packing strategies are needed to improve the predictive power of synthetic polymer simulations and overcome time scale limitations.^31,45^

#### 2D Structure and Connectivity

Building accurate polymer models remains a challenge due to the lack of dedicated tools and standardized workflows, unlike the well-established methods available for biomolecules. Many researchers repurpose building tools originally developed for biomolecular simulations, such as AMBER tLEAP,^47^ which do not inherently consider synthetic polymer-specific characteristics like tacticity, branching, or polydispersity.

Failure to rigorously prepare synthetic polymer systems often results in idealized, uniform ensembles that poorly reflect experimental conditions, potentially limiting the accuracy and relevance of simulations. The extent of accuracy loss is currently unclear, but efforts are being made by researchers to build polymer ensembles with their experimental counterparts in mind, considering the statistical distribution of properties such as polydispersity and blockiness that arise from specific polymerization methods.^48–52^

While commonly used small-molecule linear notations, such as SMILES^53^ (Simplified Molecular Input Line Entry System) strings, are effective for representing individual monomer units, they become impractical for describing full polymer chains. Single-line notations are cumbersome and inefficient when used to capture the molecular weight and structural complexity of experimentally relevant polymer systems, hindering accurate representation of chain connectivity and the statistical distributions characteristic of polymer ensembles.^54^ Attempts have been made to extend the SMILES syntax to polymer, primarily driven by a demand for input formats appropriate for use in machine learning models.^54,55^ New notations such as BIGSmiles,^56^ PSMILES,^57^ PolyGrammar,^58^ and CGsmiles^59^ aim to more effectively capture the complexity of polymer substructures at the single-chain level, enabling both compact storage and more accurate representation^60^ of the macromolecular features and distributional characteristics of polymer systems.

Building complex 2D polymer architectures, such as highly branched networks and polydisperse systems, remains a significant challenge due to the lack of versatile, widely adopted tools capable of handling diverse polymer chemistries. While some specialized tools exist such as the NIH GLYCAM carbohydrate builder for glycopolymers,^61,62^ these lack the flexibility needed for extensive functionalization or building complex topologies and are limited in scope to glycopolymers. As a result, many polymer researchers develop their own bespoke workflows, which are often poorly documented and difficult to reproduce, further exacerbating inconsistencies in polymer simulations.

#### 3D Conformation

The protein data bank (PDB),^63^ an extensive collection of 3D protein structures, and advanced structure prediction tools like AlphaFold^64^ provide extensive options for empirical and nonempirical prediction of initial structures for protein modeling.^63^ Additionally, software like Schrödinger’s Protein Preparation Wizard^65^ or PDBFixer^66^ facilitate input correction, enhancement, and refinement of this data. The PDB file format is a widely used structure file format within the protein informatics community, more recently replaced by the macromolecular crystallographic information file (mmCIF).^67^ However, when it comes to polymers, the landscape is far less standardized, with each research group and software package often employing its own conventions in PDB and structure file generation, leading to inefficiencies in data exchange and poor interoperability.

For polymers, especially amorphous ones, there is a lack of suitable 3D structure prediction methods. Researchers often rely on small-molecule conformer generation tools, such as RDKit^68^ and OpenEye Omega,^69^ to approximate polymer configurations. While these tools can generate reasonable conformations for individual repeat units or oligomers, they are designed for small molecule modeling workflows and can only process one chain at a time. This approach disregards extended chain dynamics, polydispersity, entanglement effects and the rich conformational landscape that are typical of polymer systems. Some commercial software, like BIOVIA Materials Studio,^70^ offers polymer-building capabilities, but its proprietary nature limits broader adoption. For force fields that use semiempirical or quantum mechanical (QM) models such as AM1-BCC and RESP to generate molecule charges from electrostatic potentials, particular care must be taken when generating a realistic 3D conformer, as these methods are influenced by the conformer used as an input.^46,71^

#### Building an Initial Configuration

Efforts to automate polymer structure generation and initial configuration setup face additional hurdles. Many synthetic polymers are amorphous or lack long-range order, forcing researchers to generate initial approximations of amorphous structures with varying degrees of internal order. Packing polymer chains at experimentally relevant densities remains a challenge for synthetic polymer modeling; poorly initialized systems can lead to unphysical structures and require extensive equilibration to reach realistic configurations, significantly increasing computational costs. These limitations highlight the urgent need for robust, flexible, and reproducible tools tailored specifically for polymer modeling.

#### Open-Source Polymer Building Tools

Table 1 summarizes the polymer-specific building capabilities of a range of software tools. For initial configuration generation, tools like gmxpolymer,^72^ Packmol,^73^ Polyply,^31^ PSP,^74^ and RadonPy^75^ and offer methods to pack polymer systems in preparation for Molecular Dynamics. Packmol,^73^ perhaps the most widely used, can arrange 3D molecular structures (provided by the user) into a specified box shape and position. This is sufficient for stable biopolymers with single conformations and small ligands, but the lack of options for conformation relaxation, self-avoidance, or density matching makes Packmol unsuitable for building entangled polymer systems. RadonPy^75^ arranges polymer chains randomly in the simulation cell then performs a 21-step equilibration process to reach target density and chain entanglement. Although effective for generating entangled structures, this extensive process reduces building throughput and is unsuitable for larger systems. Polymatic^43^ uses a random-walk approach, however is limited to single polymer chains, and does not enforce self-avoidance or perform packing. Polyply^31^ stands out as a more effective option for polymer packing, overcoming the pitfalls of conformer generation tools using self-avoiding walks, density control, multiple-chain packing, and energy minimization, but it is tightly integrated with GROMACS, requiring GROMACS parameter and input files. PSP^74^ takes a different approach by starting from SMILES representations, employing a random-walk packing method to generate infinite polymer chains, crystal structures, or amorphous structures with density control. However, PSP is built around LAMMPS input/output files. Lastly, gmxpolymer^72^ has recently emerged as an amorphous polymer structure generator but is also restricted to the GROMACS file system. Each tool provides valuable functionalities and specific constraints; a careful selection will depend on the simulation needs. To overcome the file format specificity which is a feature for many of these tools, software like ParmEd^76^ (no longer maintained), MDAnalysis,^77^ MDTraj,^78^ or OpenFF interchange^79^ enable flexible and robust parsing and interconversion between file formats, somewhat alleviating interoperability issues.

**Table 1:** Comparison of the Building Capabilities of Open-Source Non-Commercial Polymer Building Tools

Tool	Homopolymers	Copolymers	Branched Polymers	Tactic polymers	Polydisperse systems	Bespoke monomer input (format)	Actively maintained^b^
CHARMM Polymer Builder	Y	Y	N	Y	N	N (predefined list of monomers)	N
EMC	Y	Y	Y	Y	N	Y (monomer and terminal SMILES)	Y
mBuild	Y	Y	Y	Y	N	Y (monomer SMILES)	Y
Moltemplate	Y	Y	Y	Y	N	Y (monomer PDB or xyz)	Y
PolyCostruct	Y	Y	Y	Y	N	Y (monomer PDB or itp)	Y
Polymatic	Y	Y	Y	Y	N	Y (monomer pdb/xyz and monomer parameters)	N
Polyply	Y	Y	Y	Y	N	N^a^ (predefined library of monomers)	Y
PySIMM	Y	Y	Y	Y	Y	Y (monomer SMILES or mol file)	N
PySoftK	Y	Y	Y	Y	N	Y (monomer SMILES)	Y
RadonPy	Y	Y	Y	Y	N	Y (monomer SMILES)	Y
SwiftPol	Y	Y	Y	Y	Y	Y (monomer SMILES + Reaction SMARTS)	Y

a New monomers can be added to the library by the user.

b A tool is considered actively maintained if changes have been committed to its source code within the last 2 years.

Significant gaps remain in the ability to construct complex topologies such as cross-linked networks and branched architectures.^80,81^ While several noncommercial *in silico* tools have been developed to address specific aspects of polymer building such as PySoftK,^82^ CHARMM polymer builder,^83^ mBuild,^84^ Polyply,^31^ RadonPy,^75^ SwiftPol,^50^ Polyconstruct,^85^ Moltemplate,^86^ Enhanced Monte Carlo (EMC),^87^ PySIMM,^88^ and Polymatic,^43^ these often focus on linear polymers at the single-chain level, underrepresenting other structures and macroscale features such as polydispersity. These polymer building tools can be used to build more complex topologies, but often lack the inherent capability to do so.

Even in atomistic polymer models, coarse-graining plays a critical role in efficiently generating realistic initial configurations.^48^ By temporarily removing atomic detail during the most computationally demanding stages of equilibration, coarse-grained models can accelerate system relaxation while preserving essential large-scale features.^89^ Atomic detail can then be reinstated prior to production runs, enabling accurate, fully atomistic simulations without the cost of full equilibration at atomic resolution.^90^ This approach maximizes sampling efficiency, allowing simulations to access the time and length scales necessary to capture hallmark properties of polymer melts, such as chain entanglement, conformational flexibility, and slow relaxation dynamics, all essential for rheology studies.^91^ For instance, Kremer–Grest polymer models^92,93^ offer an effective framework for generating equilibrated configurations with realistic chain conformations, which can then be backmapped to atomistic detail for high-resolution analysis. Among the tools reviewed in Table 1. Polyply^31^ employs coarse-graining and backmapping during its self-excluding random walk process to increase the efficiency of initial coordinate generation and bypass long equilibration times.

Another solution to achieving appropriate levels of entanglement during model preparation is performing a Monte Carlo simulation prior to molecular dynamics. Tools such as Chameleon,^94^ designed specifically for polymer model generation and Cassandra,^95^ a more generalized software, enable the integration of Monte Carlo simulation into equilibration workflows. Alternatively, Self-Consistent Field Theory^96^ can be used to generate coarse-grained initial configurations using the propagator-biased chain generation algorithm,^97^ which can then be backmapped into an all-atom model.

Experimental scattering data can also inform the initial configuration and structural features of a polymer. The Computational Reverse Engineering Analysis of the Scattering Experiment (CREASE) workflow^98^ employs both a genetic algorithm and a machine learning model to generate 3D morphologies of soft materials (including polymers) fitting target scattering profiles.

### Force Field Parametrization Challenges

#### Scalability

Figure 3A shows an example of how classical atom- types may be assigned to a monomer unit. Tools such as AMBER Antechamber,^47^ CHARMM MATCH,^99^ Foyer,^100^ OPLS PolyParGen,^101^ and the Automated Force Field Topology Builder^102^ have improved the throughput of force field parametrization by automatically assigning atom types compatible with classical force fields. These tools streamline the process of atom-type assignment and reduce the need for manual intervention, which is particularly valuable for topologies with complex chemistries.^103^ While effective in assigning atom types to smaller systems, these automated approaches also have notable weaknesses for polymers.

**Figure 3:**
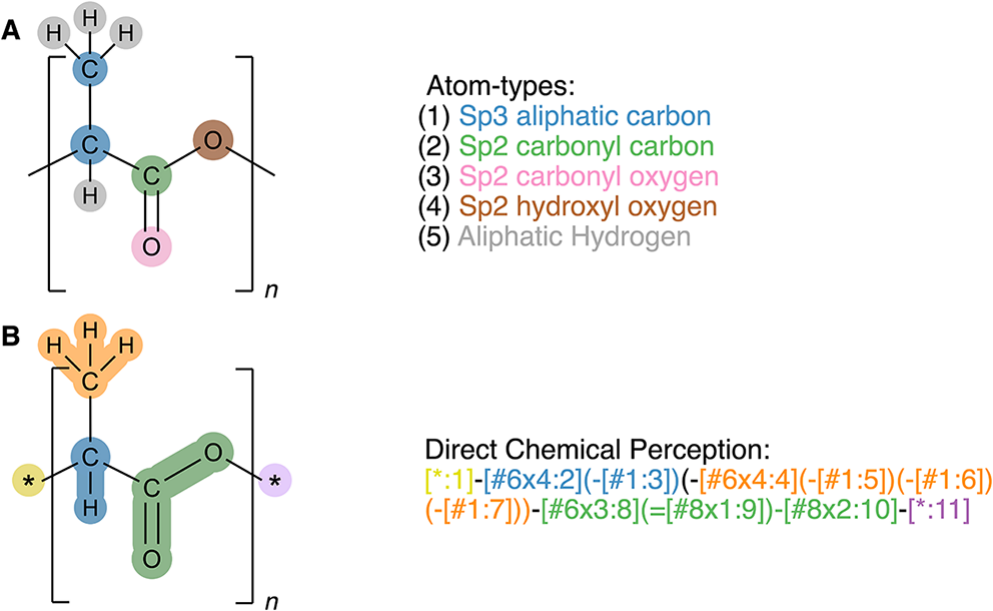
A: Manually assigned atom types for all atoms in a PLGA monomer. B: Automated atom-typing performed with direct chemical perception and the SMIRNOFF^104^ format. The resulting atom types or SMIRKS patterns are used for the mapping of bonded and nonbonded force field parameters to the study molecule, and a single pattern can encompass multiple atom types resulting in higher parametrization efficiency.

For instance, the free and open-source software AmberTools^47^ can handle both polymer building and parametrization. However, its workflows cannot always be easily automated. When preparing polymer models, researchers must usually assign atom types and parameters to the monomer unit(s) to create a “template” or “library” residue, and then map them back onto the polymer chain, as shown in Figure 3. This approach can be straightforward for common polymers but may require different degrees of manual intervention (e.g., to define polymer connectivity and ensure clear atom ordering). When dealing with complex chemistries, such as branched polymers or dendrimers, or with a very large chemical space, this approach quickly becomes time-consuming and prone to error.

Partial charges are either assigned directly from force fields in the case of OPLS or must be calculated ad-hoc for each new molecule/system in force fields such as AMBER/GAFF. In the latter case, semiempirical models (e.g., AM1-BCC) provide a cost-effective and widely used alternative to more computationally expensive quantum mechanical calculations. Though appropriate for smaller molecules, neither AM1-BCC nor more expensive DFT calculations are feasible for entire polymer chains, due to their prohibitive scaling.^105,106^ Common workflows for the parametrization of polymer partial charges involve performing QM or semiempirical calculations on oligomers, and from these, defining “template” partial charges to represent each monomer residue on the polymer chain. This process is time-consuming and generally requires some degree of manual intervention.

In summary, scalability issues are a significant limitation in the determination of partial charges in polymer systems, and therefore the calculation of electrostatic contributions to potential energy.^107^

#### Force Field Accuracy

Force field accuracy used for parametrization is an important consideration for producing a meaningful and reliable MD trajectory. The chemical diversity of synthetic polymers exceeds that of biopolymers, which are constructed from a defined set of standard building blocks.^108,109^ Although the parameters in general-purpose force fields have been shown to be appropriate to reproduce some experimental properties in synthetic polymers, e.g., density,^20^ applying classical force fields can introduce systematic errors in properties derived from the subsequent simulations,^20^ given that their fitting data sets are not representative of synthetic polymer systems.^110^ Researchers often modify force fields to quantitatively match experimental properties (e.g., *T*_g_,^111^ X-ray diffraction patterns^112^) set to a synthetic polymer, as represented in the literature survey in Figure 4. However, these ad-hoc modifications result in parameter proliferation, loss of transferability, and importantly poor reproducibility, since new parameters are often generated using different methods from those used in the original force field.^104,112,113^

**Figure 4:**
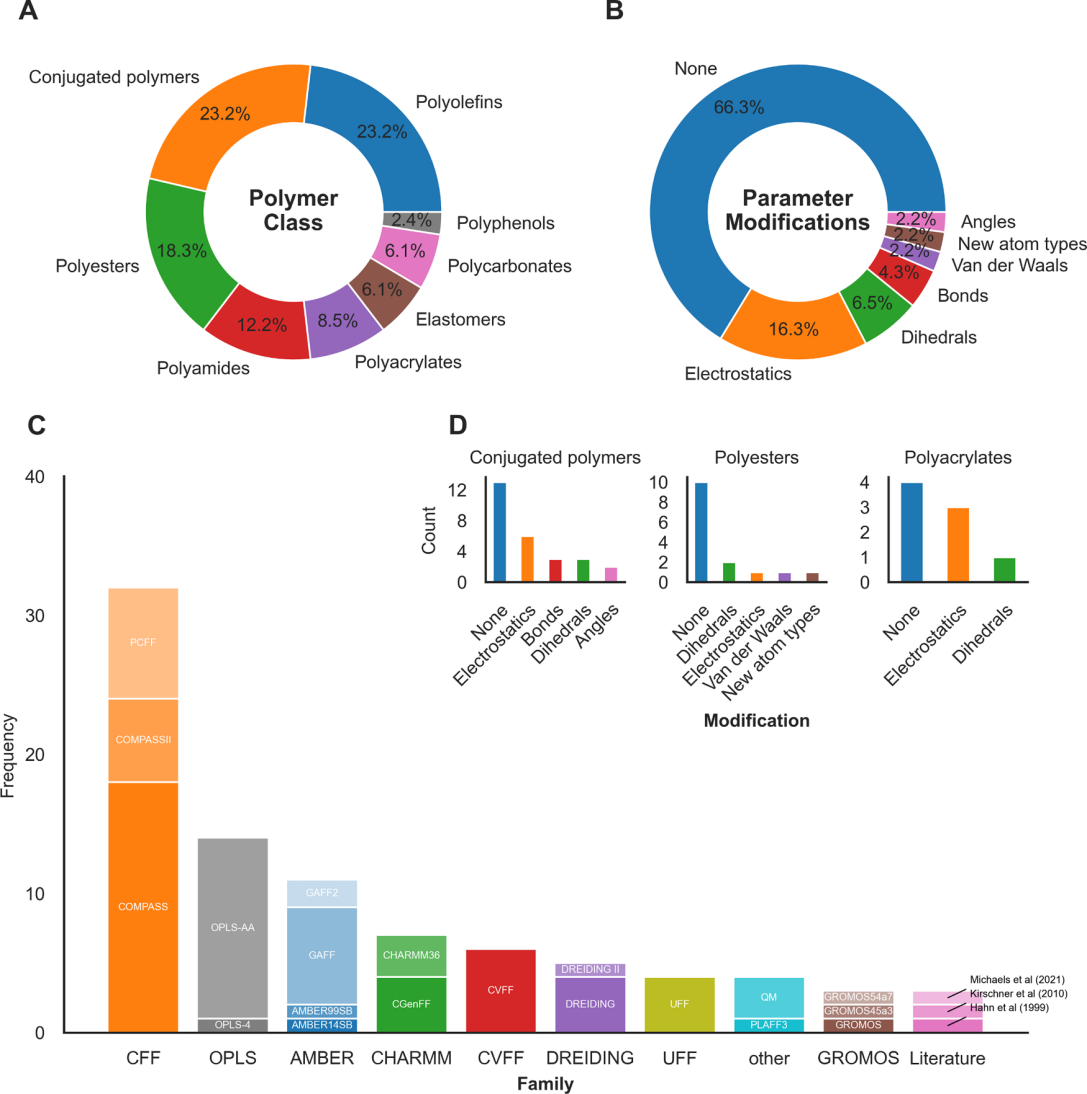
Survey of synthetic polymer modeling studies in the literature from the last 10 years, showing the range of polymer classes sampled (A), force fields used, (C) parameters modified by parameter type (B), and the parameter adjustment breakdown for the top 3 most frequently adjusted polymer classes (D). Studies using coarse-graining, reactive force fields, and polarizable force fields were excluded. Full search criteria and reference list included in Supporting Information.

For instance, the Polymer Consistent Force Field (PCFF)^114,115^ was designed to model the unique properties of polymeric materials more accurately. Despite its name and domain-specific nature, PCFF was not found to be the primary force field used in polymer MD in our literature survey (Figure 4). PCFF has not undergone the same level of continuous refinement and benchmarking as general-purpose force fields, resulting in inaccuracies for some chemistries.^116^ This leads some researchers to prefer adapting more frequently optimized, and widely published alternatives such as OPLS-AA or AMBER. Additionally, PCFF is most effectively implemented within BIOVIA Materials Studio,^70^ a proprietary software suite, which poses constraints for researchers working in open-source environments and focusing on FAIR data practices. This discrepancy further highlights the need for systematic validation and further development of open-source polymer-specific force fields to ensure accurate simulations of synthetic polymer systems.

Figure 4 illustrates the vast range of parametrization approaches found in the literature for 8 synthetic polymer classes. Our analysis reveals inconsistencies in force field usage and modification, further emphasizing the need for reproducibility and interoperability. From the 82 studies sampled, 23 different force fields were used, with additional modifications to the force field made in 27 instances. The general purpose consistent force field COMPASS^117^ is the most widely used for synthetic polymer simulation, followed by OPLS-AA.^105^ Conjugated polymer parametrization features the highest levels of intervention, with parameter modifications being made in a third of the studies. Electrostatics are overall the most frequently modified parameter.

Another common source of error in polymer force fields is the application of parameters derived from equilibrium-based models to study out-of-equilibrium conditions. While progress has been made to address this issue in coarse-grained models,^118,119^ future advances in atomistic force fields could benefit from explicitly accounting for these limitations.

#### Conjugated Polymers

Capturing the behavior of conjugated polymers using force fields is a particular challenge due to the relationship between electronic delocalization and molecular conformation.^120^ Classical force fields, which rely on fixed atomic charges and predefined bonded and nonbonded interactions, often fail to accurately represent the long-range intramolecular structural constraints and intermolecular interactions from π-conjugation effects, resulting in inaccuracies in both molecular structure and charge distributions in conjugated polymer MD systems.^121–124^ The common process of fragmenting molecules to reduce computational cost in force field fitting can potentially disrupt long-range electronic effects and conjugation, leading to a poor description of conjugated polymers.^125^ Stern et al. have proposed the use of the Wiberg Bond Order to represent the extent of preservation of conjugation systems during the fragmentation process, demonstrating the efficacy of considering WBO when generating accurate small molecule force field parameters.^121,125^

There is no standardized methodology for fitting and benchmarking force fields for conjugated polymers, resulting in a range of ad hoc parametrization strategies being applied by researchers.^126^ Many studies rely on refitting general-purpose force fields, which may not be chemically appropriate, or deriving parameters directly from QM and experimental data, often yielding highly specialized, system-specific parameter sets that lack general applicability.^112,127^

Torsion parameters are a recurring source of inconsistency in polymer force fields, particularly for conjugated systems where inter-ring torsions dictate conformational preferences. These torsions are influenced both by local steric effects and also by long-range conjugation and nonbonded interactions, making their accurate parametrization challenging.124

Compounding this issue, atomic partial charges directly impact torsional behavior.^124^ While tools such as TorsionDrive^128^ offer systematic approaches for characterizing potential energy surfaces around torsions, they do not explicitly consider extended conjugation effects. For example, different combinations of conjugated subunits exhibit profoundly different electronic properties and torsional profiles when covalently bonded,^129^ making a monomer-wise parametrization approach (common in biopolymer force fields) unsuitable.^121,125^

Combined, these issues would necessitate the refinement of both torsions and partial charges using QM methods to ensure the correct determination of the lowest energy conformer and correct subsequent parameter fitting.^126^ However, selective QM refinement results in a plethora of ad-hoc corrections to force fields, where researchers apply their preferred level of theory, charge optimization models, and fitting workflows to different degrees, inevitably leading to inconsistencies between studies, as well as force field proliferation and lack of reusability.^112,128,130,131^

### Next-generation Solutions

#### Automated Environment Perception

The SMIRNOFF force field format, first introduced by the Open force field initiative, represents a move away from traditional atom-typing methods and toward more efficient and automated approaches.^132^ By leveraging SMIRKS^133^ pattern matching, direct chemical perception can classify and identify atomic environments with precision, enabling more accurate and reproducible parametrization.^104^ Unlike SMILES strings, which offer limited structural detail, SMIRKS patterns encode rich chemical context, providing a distinct and detailed description of an atom’s environment (see Figure 3B) independently of the molecule’s embedded atom indexing and order metadata. SMIRNOFF force fields also assign chemical substructures in a hierarchical manner, with longer and more detailed SMIRKS patterns superseding the assignment of simpler single-atom substructures. This approach, dubbed “Last one wins” by Mobley et al.^104^ ensures that each substructure in the molecule has been assigned the most representative parameters.

This approach automates atom-typing with high accuracy, significantly reducing the need for manual intervention and eliminating the user bias often associated with traditional atom-typing methods. By ensuring consistent and systematic atom classification, it also addresses the issue of parameter proliferation, a common challenge in classical force fields. These advantages make this methodology indispensable for next-generation polymer force field development and parametrization workflows, effectively overcoming many of the challenges associated with applying classical atom-typing approaches to complex polymer systems. In cases where AMBER or OPLS-AA force field parameters are preferred, Polymer Structure Predictor,^74^ PolyParGen^101^ GUI, and foyer^100^ have been developed to provide defined workflows for atom-typing for polymers, removing the need for user intervention.

#### Machine-Learned Force Field Parameters

The emergence of machine learning for the estimation of force field parameters offers an opportunity to completely shift away from traditional atom-typing and parametrization approaches.^134^ Recently developed methods employ neural networks (NNs) trained to predict critical molecular parameters such as atomic charges, bond force constants, and torsional potentials, directly from the molecular graph, significantly reducing the need for manual intervention.^135,136^ Improved computational hardware has led to the generation of extensive QM data sets,^137^ which can be used to train more well-informed neural network potentials to predict force field parameters, enabling the generation of application-specific force fields in a fraction of the time taken for the equivalent QM work.^138^ We note that neural network potentials developed for the direct prediction of the potential energy surfaces (beyond the scope of this Perspective) have established themselves as a potential avenue to remove the need for force field parameters altogether.^139,140^

The Grappa framework^141^ uses a NN to assign bonded parameters based on a system’s molecular graph, with partial charges calculated at the semiempirical AM1-BCC level. While its validation data set primarily focuses on biopolymers, its demonstrated scalability and transferability suggest it can also be applied effectively to synthetic polymers.

NNs such as Espaloma^136^ (Extensible Self-Parameterized Learning of Molecular Attributes) and NAGL^135^ (Neural Atomistic Graph Learner) have been integrated into polymer parametrization workflows, showing performance comparable to semiempirical methods while easing the reliance on semiempirical calculations at the point of use.^107,142–145^ These NNs are trained to predict entire parameter sets, including AM1-BCC partial charges.

While the aforementioned models use semiempirical AM1-BCC charges as their reference, NNs using a higher level of theory in their training sets will be expected to provide charges that better represent the electronic structure. For example, DASH^146^ (Dynamic Attention-Based Substructure Hierarchy) a partial charge graph neural network trained to predict QM charges, and NAGL-MBIS, which uses the same graph convolutional network architecture as NAGL to reproduce QM MBIS charges.^147^

However, the importance of critically evaluating and interrogating the architecture, training process, and the output of NN force fields to ensure user confidence cannot be overstated.^148^ Promoting explainable NNs is essential to gaining a clear understanding of how parameters are assigned, enhancing confidence in simulation outputs.^149^ Additionally, the rapid dissemination of NN force fields without thorough validation carries similar risks associated with classical force fields that we have discussed in this perspective.^150^

#### Ad hoc Parameter Generation

Where pre-existing force field parameters or pretrained NNPs do not properly capture edge cases in a synthetic polymer system, ad hoc force field fitting software such as OpenFF BespokeFit,^151^ The Force Field Toolkit,^152^ and AMBER Paramfit^153^ offer a robust solution for generating high-quality, molecule-specific parameters. Originally developed to improve the accuracy of therapeutic ligand parametrization, these tools take a holistic approach to the generation of new force field parameters tailored to specific molecules or fragments in the system, using quantum chemical (QC) data to ensure a high level of accuracy. The Topology Automated Force-field Interactions (TAFFI) framework^154^ implements this approach for polymers, by automating the integration of quantum chemistry data into existing force fields, extending parameter sets to effectively cover the required chemical space.

By leveraging this integration of QC data, ad hoc fitting tools have the potential to overcome unreproducible parameter tweaking for unique synthetic polymers or chemistries that fall outside the scope of general-purpose force fields, such as those containing rare or novel functional groups.

#### Better Benchmarking and Data Sharing

The lack of standardized benchmarking for force fields and building tools is a well-recognized weakness that limits their accuracy and transferability.^155^ Synthetic polymer force field benchmarking lags particularly far behind that of other domains, largely due to the absence of standardized experimental and simulation data sets and their reuse.^156^ Existing data repositories provide extensive coverage of biopolymers such as the PDB,^63^ LIPID MAPS structure database,^157^ open-source protein–ligand benchmark data sets,^158,159^ MDverse,^3^ MDDB.^2^ Small drug-like molecules are well represented in OMol25,^137^ ChEMBL,^160^ and the Cambridge Crystallographic Data Centre.^161^ The materials science community have also made strides in open-source data repositories, with the NOMAD FAIR research data archive,^162^ the Materials Research Project,^163^ and the Open Molecular Crystals 2025 data set.^164^ Including synthetic polymers and other soft materials in these data repositories could unlock new possibilities for developing and benchmarking transferable and standardized force fields, bridging a critical gap in the field.^165^

While synthetic polymer force field benchmarking studies have been performed,^18,75,166–168^ this is not to the same extent seen for as biopolymers; the experimental data sets used during testing are rarely published in full, and not widely reused. To advance polymer simulations, polymer scientists could adopt the same paradigms used in biomolecular simulations, and force field benchmarking,^169,170^ developing FAIR-compliant^1^ shared, reusable data sets,^2,159,171^ and establishing community-driven benchmarks.^172^

Efforts like the Community Resource for Innovation in Polymer Technology (CRIPT)^173^ and CAMPARI^174^ promote open-source and FAIR data sharing in polymer science, offering a novel approach to describing hierarchical structures present in polymers and providing a blueprint for the organization and storage of associated data, including computational experiments.^173^ However, although this infrastructure exists for efficient data storage and reuse, limited adoption by the community poses a barrier to its population and advancement, particularly with quantifying uncertainty in the available data.^175^ Prioritizing standardized and openly shared simulation data could unlock transformative advances in the predictive design of functional polymeric materials, similarly to those observed in biopolymer design.

## Summary and Outlook

In this perspective, we have examined the challenges and inconsistencies that arise in the parametrization and building of polymers using classical force fields. A central theme of our discussion is the inherent pitfalls of established “legacy” methods, where oversimplified assumptions often overlook the unique structural and dynamic complexity of polymers. The lack of a shared knowledge base and accepted standards in the polymer modeling community (if one can be defined) has led to limitations and systematic issues, especially for conjugated polymers. Existing workflows struggle to accommodate the diversity of chemical environments in synthetic polymers, presenting barriers to large-scale polymer informatics workflows.

Despite these hurdles, promising new tools are emerging that better capture unique topologies, and diverse chemistries while affording an improved representation of their electronic properties. We have here proposed a new approach (Figure 2) to streamline MD simulation building via direct chemical perception and machine learning potentials, shifting away from human-intensive, error-prone, and computationally expensive approaches. We note that, to promote the adoption of new tools by the polymer modeling community, we must ensure that they (i) have good interoperability, comprehensive documentation, (ii) are actively maintained, and (iii) do not require extensive specialized knowledge of their dependencies.

Looking ahead, we can draw valuable lessons from the biomolecular simulation community, where parametrization tools and techniques have been honed over decades. The dissemination of these techniques and their associated benchmarks has maintained momentum in the progress of simulating biopolymers that cannot be observed in synthetic polymer modeling.

A core consideration moving forward is the fostering of greater knowledge sharing within the polymer modeling community. By aligning methods and best practices and building on each other’s successes we can address the current shortcomings in polymer modeling, toward the next generation of polymer research and applications.

## Supplementary Material



## Notes

Literature search criteria and citations for papers featured in Figure 4 (PDF)
