# Supplementary material for: Atomistic Polymer Modeling: Recent Advances and Challenges in Building and Parametrization Workflows
Source: Macromolecules. 2025 Oct 28;58(21):11509–22. doi: 10.1021/acs.macromol.5c01166 (PMC12613811; doi:10.1021/acs.macromol.5c01166)
Supplement: Supplementary file 1 [file ma5c01166_si_001.pdf]

Supporting Information

Atomistic polymer modeling: recent advances and challenges in building and parameterization workflows

*Hannah N. Turney, Micaela Matta**

Department of Chemistry, King's College London Strand Campus (East Wing), London, WC2R 2LS, United Kingdom

*micaela.matta@kcl.ac.uk

Literature search criteria for Figure 3.[1–82]

- Web of Science
- Survey performed between 12/29/24 to 05/15/25
- <name of polymer or polymer class> 'Molecular Dynamics'

Table S1. Search terms used to find molecular dynamics studies for each polymer class

**Polymer Class**	**Search terms**
Conjugated Polymers	Polythiophene<br>PEDOT<br>Polypyrrole/PPY<br>Poly NDI/Poly Naphthalenediimide<br>Phenyleneethynylene<br>Polyaniline/PANI<br>Diketopyrrolopyrrole/DPP
Polyolephins	Polyethylene<br>Polybutylene<br>Polypropylene
Polyamides	Polyamide<br>Nylon
Polyphenols	Polyphenol
Elastomers	Silicone<br>Rubber
Polyesters	Polylactide-co-glycolide<br>Polylactide<br>Polycaprolactone<br>Polyethylene terephthalate
Polyacrylates	Polymethyl methacrylate<br>Polyacrylate<br>Polyacrylic acid
Polycarbonates	Polycarbonate

Exclusion criteria:

- Papers older than 10 years
- Coarse grained simulations
- Reactive force field simulations
- Polarizable force field simulations
- Polyphenol simulations studying low molecular weight polyphenols (< 3 monomers), common in plants.

Full literature survey bibliography:

(1) Stipa, P.; Marano, S.; Galeazzi, R.; Minnelli, C.; Mobbili, G.; Laudadio, E. Prediction of Drug-Carrier Interactions of PLA and PLGA Drug-Loaded Nanoparticles by Molecular Dynamics Simulations. *Eur. Polym. J.* **2021**, *147*, 110292. https://doi.org/10.1016/j.eurpolymj.2021.110292.

(2) Sharma, A.; Sharma, S. A Molecular Dynamics Study of Adhesion of Polyvinyl-Chloride Coatings to the Aluminum Surface. *IOP Conf. Ser. Mater. Sci. Eng.* **2022**, *1248* (1), 012062. https://doi.org/10.1088/1757-899X/1248/1/012062.

(3) Di, J.; Lei, Z.; Rui, M.; Bai, R.; Xia, P.; Yan, C. Interfacial Mechanical Properties of Graphene on Randomly Rough PET Substrate Surface: A Molecular Dynamics Study. *Appl. Surf. Sci.* **2024**, *648*, 159109. https://doi.org/10.1016/j.apsusc.2023.159109.

(4) Zheng, J.; Wang, D.; Zhang, Q.; Song, M.; Jiao, M.; Zhang, Z. Molecular Dynamics Simulation and Structure Changes of Polyester in Water and Non-Aqueous Solvents. *Materials* **2022**, *15* (6), 2148. https://doi.org/10.3390/ma15062148.

(5) Sangkhawasi, M.; Remsungnen, T.; Vangnai, A. S.; Poo-arporn, R. P.; Rungrotmongkol, T. All-Atom Molecular Dynamics Simulations on a Single Chain of PET and PEV Polymers. *Polymers* **2022**, *14* (6), 1161. https://doi.org/10.3390/polym14061161.

(6) Yungerman, I.; Starodumov, I.; Fulati, A.; Uto, K.; Ebara, M.; Moskovitz, Y. Full-Atomistic Optimized Potentials for Liquid Simulations and Polymer Consistent Force Field Models for Biocompatible Shape-Memory Poly(ε-Caprolactone). *J. Phys. Chem. B* **2022**, *126* (21), 3961–3972. https://doi.org/10.1021/acs.jpcb.2c01973.

(7) Du, X.; Jasim, D. J.; Sajadi, S. M.; Hekmatifar, M.; Salahshour, S.; Sabetvand, R.; Arefpour, A.; Toghraie, D. The Molecular Dynamics Description of Polycaprolactone Coating Effect on Mechanical Behavior of Polycaprolactone/BG-AK Bio-Nanocomposites. *Int. J. Adhes. Adhes.* **2024**, *129*, 103577. https://doi.org/10.1016/j.ijadhadh.2023.103577.

(8) McAliley, J. H.; Bruce, D. A. Development of Force Field Parameters for Molecular Simulation of Polylactide. *J. Chem. Theory Comput.* **2011**, *7* (11), 3756–3767. https://doi.org/10.1021/ct200251x.
(9) Lange, J.; de Souza junior, F. G.; Nele, M.; Tavares, F. W.; Segtovich, I. S. V.; da Silva, G. C. Q.; Pinto, J. C. Molecular Dynamic Simulation of Oxaliplatin Diffusion in Poly(Lactic Acid-Co-Glycolic Acid). Part A: Parameterization and Validation of the Force-Field CVFF. *Macromol. Theory Simul.* **2016**, *25* (1), 45–62. https://doi.org/10.1002/mats.201500049.
(10) Modi, V.; Karttunen, A. J. Molecular Dynamics Simulations on the Elastic Properties of Polypropylene Bionanocomposite Reinforced with Cellulose Nanofibrils. *Nanomaterials* **2022**, *12* (19), 3379. https://doi.org/10.3390/nano12193379.
(11) Cai, M.; He, X.; Liu, B. Revealing the Effect of the Molecular Weight Distribution on the Chain Diffusion and Crystallization Process under a Branched Trimodal Polyethylene System. *Polymers* **2024**, *16* (2), 265. https://doi.org/10.3390/polym16020265.
(12) Hafezi, M.-J.; Sharif, F. Dynamic Crosslinking of Polyethylene by Hydrogen Bonding Grafted Motifs: A Molecular Dynamics Simulation Study. *Macromol. Chem. Phys.* **2023**, *224* (23), 2300163. https://doi.org/10.1002/macp.202300163.
(13) Olowookere, F. V.; Al Alshaikh ,Ali; Bara ,Jason E.; and Turner, C. H. Effects of Chain Length on the Structure and Dynamics of Polyvinyl Chloride during Atomistic Molecular Dynamics Simulations. *Mol. Simul.* **2023**, *49* (15), 1401–1412. https://doi.org/10.1080/08927022.2023.2234493.
(14) Jagarlapudi, S. S.; Cross, H. S.; Das, T.; Goddard, W. A. I. Thermomechanical Properties of Nontoxic Plasticizers for Polyvinyl Chloride Predicted from Molecular Dynamics Simulations. *ACS Appl. Mater. Interfaces* **2023**, *15* (20), 24858–24867. https://doi.org/10.1021/acsami.3c02354.
(15) Huang, W.; Geng, X.; Liu, Z.; Zhou, C. Molecular Dynamics Study of Polymeric Stabilizers as Soil Improvement Materials. *Chem. Phys. Lett.* **2022**, *806*, 139985. https://doi.org/10.1016/j.cplett.2022.139985.
(16) Feng, Y.; Wang, W.; Wang, S. PVA Fiber/Cement-Based Interface in Silane Coupler KH560 Reinforced High Performance Concrete – Experimental and Molecular Dynamics Study. *Constr. Build. Mater.* **2023**, *395*, 132184. https://doi.org/10.1016/j.conbuildmat.2023.132184.
(17) He, C.; Xu, B.; Li, X. Effects of Modified Single-Wall Carbon Nanotubes on the Mechanical Properties of Polyvinyl Alcohol Composites by Molecular Dynamics Simulation. *Mater. Today Commun.* **2023**, *35*, 105598. https://doi.org/10.1016/j.mtcomm.2023.105598.
(18) Su, Y.; Lv, H.; Feng, C.; Zhang, C. Hydrogen Permeability of Polyamide 6 as the Liner Material of Type Ⅳ Hydrogen Storage Tanks: A Molecular Dynamics Investigation. *Int. J. Hydrog. Energy* **2024**, *50*, 1598–1606. https://doi.org/10.1016/j.ijhydene.2023.10.154.
(19) Chantawansri, T. L.; Yeh, I.-C.; Hsieh, A. J. Investigating the Glass Transition Temperature at the Atom-Level in Select Model Polyamides: A Molecular Dynamics Study. *Polymer* **2015**, *81*, 50–61. https://doi.org/10.1016/j.polymer.2015.09.069.
(20) Rosenauer, P.; Kratzer, C.; Larisegger, S.; Radl, S. Extraction of Mechanical Parameters via Molecular Dynamics Simulation: Application to Polyimides. *Polymers* **2024**, *16* (6), 813. https://doi.org/10.3390/polym16060813.

(21) Abdul Manap, A. H.; Shamsuddin, L.; Mohamed, K. The Study of Polydimethylsiloxane Nanocone Distortion in the Demolding Process Using Molecular Dynamics Method. *AIP Adv.* **2022**, *12* (4), 045011. https://doi.org/10.1063/5.0078072.
(22) Abdul Manap, A. H.; Md Izah, S. S.; Mohamed, K. Molecular Dynamics Study of Poly(Dimethylsiloxane) Nanostructure Distortion in a Soft Lithography Demolding Process. *ACS Omega* **2019**, *4* (23), 20257–20264. https://doi.org/10.1021/acsomega.9b02547.
(23) Gao, P.; Pu, W.; Wei, P.; Kong, M. Molecular Dynamics Simulations on Adhesion Energy of PDMS-Silica Interface Caused by Molecular Structures and Temperature. *Appl. Surf. Sci.* **2022**, *577*, 151930. https://doi.org/10.1016/j.apsusc.2021.151930.
(24) Fujimoto, K.; Ishikawa, H.; Tang, Z.; Okazaki, S. All-Atom Molecular Dynamics Study of the Impact Fracture of Glassy Polymers. III: Compressive Fracture of PC and PMMA. *Polymer* **2023**, *283*, 126276. https://doi.org/10.1016/j.polymer.2023.126276.
(25) Monk, J. D.; Haskins, J. B.; Bauschlicher, C. W.; Lawson, J. W. Molecular Dynamics Simulations of Phenolic Resin: Construction of Atomistic Models. *Polymer* **2015**, *62*, 39–49. https://doi.org/10.1016/j.polymer.2015.02.003.
(26) Bone, M. A.; Macquart, T.; Hamerton, I.; Howlin, B. J. A Novel Approach to Atomistic Molecular Dynamics Simulation of Phenolic Resins Using Symthons. *Polymers* **2020**, *12* (4), 926. https://doi.org/10.3390/polym12040926.
(27) Zhao, D.; Kim, D.; Ghosh, S.; Wang, G.; Huang, W.; Zhu, Z.; Marks, T. J.; Zozoulenko, I.; Facchetti, A. Mechanical, Morphological, and Charge Transport Properties of NDI Polymers with Variable Built-in Π-Conjugation Lengths Probed by Simulation and Experiment. *Adv. Funct. Mater.* **2024**, *34* (4), 2310071. https://doi.org/10.1002/adfm.202310071.
(28) Zanetti-Polzi, L.; Djemili, R.; Durot, S.; Heitz, V.; Daidone, I.; Ventura, B. Allosteric Control of Naphthalene Diimide Encapsulation and Electron Transfer in Porphyrin Containers: Photophysical Studies and Molecular Dynamics Simulation. *Chem. – Eur. J.* **2020**, *26* (72), 17514–17524. https://doi.org/10.1002/chem.202003151.
(29) Tang, C. G.; Syafiqah, M. N.; Koh, Q.-M.; Ang, M. C.-Y.; Choo, K.-K.; Sun, M.-M.; Callsen, M.; Feng, Y.-P.; Chua, L.-L.; Png, R.-Q.; Ho, P. K. H. Water Binding and Hygroscopicity in π-Conjugated Polyelectrolytes. *Nat. Commun.* **2023**, *14* (1), 3978. https://doi.org/10.1038/s41467-023-39215-9.
(30) Tadesse, M. Y.; Marioni, N.; Zhang, Z.; Ganesan, V. Influence of Cation and Anion Chemistry on the Ionic Conductivity and Transference Number of Zwitterionic Polymer-Supported Ionic Liquid Electrolytes. *ACS Appl. Energy Mater.* **2025**, *8* (6), 3314–3326. https://doi.org/10.1021/acsaem.4c02650.
(31) Alsaedi, M. K.; Tadesse, M. Y.; Ganesan, V.; Panzer, M. J. Zwitterionic Polymer Ionogel Electrolytes Supported by Coulombic Cross-Links: Impacts of Alkali Metal Cation Identity. *J. Phys. Chem. B* **2024**, *128* (13), 3273–3281. https://doi.org/10.1021/acs.jpcb.3c08144.
(32) Behbahani, A. F.; Allaei, S. M. V.; Motlagh, G. H.; Eslami, H.; Harmandaris, V. A. Structure and Dynamics of Stereo-Regular Poly(Methyl-Methacrylate) Melts through Atomistic Molecular Dynamics Simulations. *Soft Matter* **2018**, *14* (8), 1449–1464. https://doi.org/10.1039/C7SM02008B.
(33) Kunche, L.; Natarajan, U. Structure and Dynamics of an Aqueous Solution Containing Poly-(Acrylic Acid) and Non-Ionic Surfactant Octaethylene Glycol n-

Decyl Ether (C10E8) Aggregates and Their Complexes Investigated by Molecular Dynamics Simulations. *Soft Matter* **2021**, *17* (3), 670–687. https://doi.org/10.1039/D0SM01322F.
(34) Hosoya, R.; Morita, H.; Nakajima, K. Analysis of Nanomechanical Properties of Polyethylene Using Molecular Dynamics Simulation. *Macromolecules* **2020**, *53* (15), 6163–6172. https://doi.org/10.1021/acs.macromol.0c01009.
(35) Sharudin, R. W.; Md Azmi, N. S.; Hanizan, A.; Akhbar, S.; Ahmad, Z.; Ohshima, M. Dynamic Molecular Simulation of Polyethylene/Organoclay Nanocomposites for Their Physical Properties and Foam Morphology. *Materials* **2023**, *16* (8), 3122. https://doi.org/10.3390/ma16083122.
(36) Saleh, A. H.; Malfreyt, P.; Sahihi, M. Deciphering Amino Acid Adsorption on PVC Surface: Insights from Molecular Dynamics and PMF Calculations. *New J. Chem.* **2024**, *48* (41), 17822–17830. https://doi.org/10.1039/D4NJ02730B.
(37) Tokhadzé, N.; Sahnoune, M.; Devémy, J.; Dequidt, A.; Goujon, F.; Chennell, P.; Sautou, V.; Malfreyt, P. Insulin Adsorption onto PE and PVC Tubings. *ACS Appl. Bio Mater.* **2022**, *5* (6), 2567–2575. https://doi.org/10.1021/acsabm.2c00029.
(38) Mintis, D. G.; Mavrantzas, V. G. Effect of pH and Molecular Length on the Structure and Dynamics of Short Poly(Acrylic Acid) in Dilute Solution: Detailed Molecular Dynamics Study. *J. Phys. Chem. B* **2019**, *123* (19), 4204–4219. https://doi.org/10.1021/acs.jpcb.9b01696.
(39) Mao, Y.-F.; Long, S.-N.; Li, Z.; Tao, W.-Q. Diffusion Behavior of VOC Molecules in Polyvinyl Chloride Investigated by Molecular Dynamics Simulation. *Int. J. Environ. Res. Public. Health* **2023**, *20* (4), 3235. https://doi.org/10.3390/ijerph20043235.
(40) Olowookere, F. V.; and Turner, C. H. Predicting Optimal Chain Lengths in Atomistic Simulations of Solvated Polymers. *Mol. Simul.* **2024**, *50* (11), 687–695. https://doi.org/10.1080/08927022.2024.2341964.
(41) Nagumo, R.; Shibata, A.; Taniguchi, I.; Iwata, S. Molecular Dynamics Simulation of the Relationship between Hydration and Water Mobilities around Piperazine-Immobilized Polyvinyl Alcohol Membranes for CO2 Capture. *Polym. J.* **2024**, *56* (10), 933–938. https://doi.org/10.1038/s41428-024-00936-3.
(42) Sun, H.; Feng, Y.; Lei, B.; Yin, X.; Wu, B. Molecular Simulation of the Effects of Water on the Interface of Plant Fiber/Polyvinyl Alcohol (PVA) Composites Prepared by Dry Preparation Method (DPM). *J. Mol. Liq.* **2023**, *391*, 123174. https://doi.org/10.1016/j.molliq.2023.123174.
(43) Zhou, J.; Qin, Z. Structure–Mechanics Relationship of Hybrid Polyvinyl Alcohol-Collagen Composite by Molecular Dynamics Simulations. *MRS Bull.* **2023**, *48* (4), 332–341. https://doi.org/10.1557/s43577-022-00416-0.
(44) Liu, Y.; Su, J.; Duan, F.; Cui, X.; Yan, W.; Jin, L. Molecular Simulation of Enhanced Separation of Humid Air Components Using GO–PVA Nanocomposite Membranes under Differential Pressures. *Phys. Chem. Chem. Phys.* **2022**, *24* (27), 16442–16452. https://doi.org/10.1039/D2CP01411D.
(45) Sahihi, M.; Fayon, P.; Nauton, L.; Goujon, F.; Devémy, J.; Dequidt, A.; Hauret, P.; Malfreyt, P. Probing Enzymatic PET Degradation: Molecular Dynamics Analysis of Cutinase Adsorption and Stability. *J. Chem. Inf. Model.* **2024**, *64* (10), 4112–4120. https://doi.org/10.1021/acs.jcim.4c00079.
(46) Polêto, M.; Lemkul, J. Structural and Electronic Properties of Polyethylene Terephthalate (PET) from Polarizable Molecular Dynamics Simulations. ChemRxiv September 4, 2024. https://doi.org/10.26434/chemrxiv-2024-dwhb8.

(47) Berselli, A.; Menziani, M. C.; Muniz-Miranda, F. Structure and Energetics of PET-Hydrolyzing Enzyme Complexes: A Systematic Comparison from Molecular Dynamics Simulations. *J. Chem. Inf. Model.* **2024**, *64* (21), 8236–8257. https://doi.org/10.1021/acs.jcim.4c01369.
(48) Jerves, C.; Neves, R. P. P.; Ramos, M. J.; da Silva, S.; Fernandes, P. A. Reaction Mechanism of the PET Degrading Enzyme PETase Studied with DFT/MM Molecular Dynamics Simulations. *ACS Catal.* **2021**, *11* (18), 11626–11638. https://doi.org/10.1021/acscatal.1c03700.
(49) Jerves, C.; Neves, R. P. P.; Ramos, M. J.; da Silva, S.; Fernandes, P. A. Reaction Mechanism of the PET Degrading Enzyme PETase Studied with DFT/MM Molecular Dynamics Simulations. *ACS Catal.* **2021**, *11* (18), 11626–11638. https://doi.org/10.1021/acscatal.1c03700.
(50) Shah, T.; Stefan, M. C.; Torabifard, H. Dynamics of Amphiphilic Poly(ε-Caprolactone) Micelles with Doxorubicin and Transition Temperature Predictions Using All-Atom Molecular Dynamics Simulation. *J. Phys. Chem. B* **2024**, *128* (48), 11981–11991. https://doi.org/10.1021/acs.jpcb.4c05368.
(51) Sohrabian, M.; Vaseghi, M.; Ranjbar Eslamloo, S.; Sameezadeh, M.; Arab, B.; Moradi, F. Molecular Dynamics Study on Mechanical Properties of Polycaprolactone/Bioactive Glass Nanocomposites. *Comput. Mater. Sci.* **2024**, *243*, 113098. https://doi.org/10.1016/j.commatsci.2024.113098.
(52) Lightfoot, J. C.; Castro-Dominguez, B.; Buchard, A.; Parker, S. C. A Molecular Dynamics Approach to Modelling Oxygen Diffusion in PLA and PLA Clay Nanocomposites. *Mater. Adv.* **2023**, *4* (10), 2281–2291. https://doi.org/10.1039/D3MA00158J.
(53) Chehrazi, E. Molecular Dynamics Simulations of Gas Transport Properties in Cross-Linked Polyamide Membranes: Tracing the Morphology and Addition of Silicate Nanotubes. *ACS Omega* **2024**, *9* (31), 33425–33436. https://doi.org/10.1021/acsomega.3c10108.
(54) Kawabata, Y.; Gonzales, R. R.; Nakagawa, K.; Shintani, T.; Matsuyama, H.; Fujimura, Y.; Kawakatsu, T.; Yoshioka, T. Molecular Dynamics Study on the Elucidation of Polyamide Membrane Fouling by Nonionic Surfactants and Disaccharides. *Phys. Chem. Chem. Phys.* **2021**, *23* (36), 20313–20322. https://doi.org/10.1039/D1CP01455B.
(55) Li, X.; Qiu ,Zhengjie; Wu ,Yonglong; Li ,Feilong; and Zhang, R. Molecular Dynamics Study on Friction of Polymer Material Polyamide 6 (PA 6). *Mol. Simul.* **2024**, *50* (2), 89–103. https://doi.org/10.1080/08927022.2023.2276290.
(56) Yang, Q.; Li, W.; Stober, S. T.; Burns, A. B.; Gopinadhan, M.; Martini, A. Molecular Dynamics Simulation of the Stress–Strain Behavior of Polyamide Crystals. *Macromolecules* **2021**, *54* (18), 8289–8302. https://doi.org/10.1021/acs.macromol.1c00974.
(57) Ghahramani, N.; Rahmati, M. The Effect of the Molecular Weight and Polydispersity Index on the Thermal Conductivity of Polyamide 6: A Molecular Dynamics Study. *Int. J. Heat Mass Transf.* **2020**, *154*, 119487. https://doi.org/10.1016/j.ijheatmasstransfer.2020.119487.
(58) Oh, T. H.; Shin, D. S.; Im, Y.; Seo, Y.; Nathanael, A. J.; Kim, Y. J.; Jeon, J. H.; Kim, K. H.; Jung, J.-H.; Choi, I. S. Experimental and Molecular Dynamics Studies on Tensile Properties of Nylon 6/Graphene Composite Filaments. *Fibers Polym.* **2022**, *23* (6), 1684–1691. https://doi.org/10.1007/s12221-022-4864-y.

(59) Wang, Z.; Du, M.; Fang, H.; Zhao, P.; Yao, X.; Zhu, L.; Wu, Y. A Molecular Dynamics Simulation on the Influences of PDMS on the Glass Transition Temperature and the Tensile Properties of Polyaspartate Polyurea. *Polymer* **2024**, *300*, 127016. https://doi.org/10.1016/j.polymer.2024.127016.
(60) Lou, W.; Xie, C.; Guan, X. Molecular Dynamic Study of Radiation-Moisture Aging Effects on the Interface Properties of Nano-Silica/Silicone Rubber Composites. *Npj Mater. Degrad.* **2023**, *7* (1), 1–13. https://doi.org/10.1038/s41529-023-00351-8.
(61) Qian, Y.; Guo, M.; Li, C.; Bi, K.; Chen, Y. New Insight on the Interface between Polythiophene and Semiconductors via Molecular Dynamics Simulations. *ACS Appl. Mater. Interfaces* **2019**, *11* (33), 30470–30476. https://doi.org/10.1021/acsami.9b09742.
(62) Romagnoli, N.; Padula, D. Genesis of the Chirality of Polythiophene Aggregates from Classical Molecular Dynamics. *J. Phys. Chem. C* **2024**, *128* (46), 19901–19911. https://doi.org/10.1021/acs.jpcc.4c06076.
(63) Craig, B.; Skylaris, C.-K.; de Leon, C. P.; Kramer, D. *Ab Initio* Molecular Dynamics Study of AlCl4− Adsorption on PEDOT Conducting Polymer Chains. *Energy Rep.* **2021**, *7*, 111–119. https://doi.org/10.1016/j.egyr.2021.02.035.
(64) Sarrami, F.; Gueskine, V.; Zozoulenko, I. Electrochemical Oxygen Reduction Reaction at Conductive Polymer PEDOT: Insight from Ab Initio Molecular Dynamics Simulations. *Chem. Phys.* **2021**, *551*, 111308. https://doi.org/10.1016/j.chemphys.2021.111308.
(65) Lansac, Y.; Choi, C.; Jang, Y. H. Stretchable Conducting Polymer PEDOT:PSS Treated with Hard-Cation-Soft-Anion Ionic Liquid Designed from Molecular Modeling. *Bull. Korean Chem. Soc.* **2024**, *45* (11), 896–905. https://doi.org/10.1002/bkcs.12908.
(66) Floris, P. S.; Zahabi, N.; Zozoulenko, I.; Rurali, R. Anisotropic Lattice Thermal Conductivity in Highly Ordered PEDOT Fibers. *Macromol. Mater. Eng.* **2024**, *309* (10), 2400092. https://doi.org/10.1002/mame.202400092.
(67) de Izarra, A.; Choi, C.; Jang, Y. H.; Lansac, Y. Molecular Dynamics of PEDOT:PSS Treated with Ionic Liquids. Origin of Anion Dependence Leading to Cation Design Principles. *J. Phys. Chem. B* **2021**, *125* (30), 8601–8611. https://doi.org/10.1021/acs.jpcb.1c02445.
(68) Kim, D.; Franco-Gonzalez, J. F.; Zozoulenko, I. How Long Are Polymer Chains in Poly(3,4-Ethylenedioxythiophene):Tosylate Films? An Insight from Molecular Dynamics Simulations. *J. Phys. Chem. B* **2021**, *125* (36), 10324–10334. https://doi.org/10.1021/acs.jpcb.1c04079.
(69) Zhao, X.; Liu, X.; Fan, B.; Zheng, X. Optimized Anticorrosion of Polypyrrole Coating by Inverted-Electrode Strategy: Experimental and Molecular Dynamics Investigations. *Polymers* **2022**, *14* (7), 1356. https://doi.org/10.3390/polym14071356.
(70) Luo, M.; Yin, Q.; Jiang, B.; Zhou, G. Molecular Simulation Study on Electronic Property and Thermal Conductivity of Graphyne/Polypyrrole Composite. *Macromol. Theory Simul.* **2022**, *31* (4), 2100093. https://doi.org/10.1002/mats.202100093.
(71) Luo, M.; Qiao, Y.; Wang, Y.; Yin, Q.; Zhou, G. Molecular Simulation Study on Permeation Behavior of Small Molecules in Graphyne/Polypyrrole Mixed Matrix Membrane. *Polym. Compos.* **2023**, *44* (12), 8974–8987. https://doi.org/10.1002/pc.27751.

(72) Xu, Y.; Zheng, D.; Ji, W.; Abu-Zahra, N.; Qu, D. A Molecular Dynamics Study of the Binding Effectiveness between Undoped Conjugated Polymer Binders and Tetra-Sulfides in Lithium–Sulfur Batteries. *Compos. Part B Eng.* **2021**, *206*, 108531. https://doi.org/10.1016/j.compositesb.2020.108531.
(73) Fukuhima, A.; Uematsu, H. Interfacial Adhesion of Polycarbonate to Graphene and Silicon Oxide: A Comparative Molecular Dynamics Analysis. *Surf. Interfaces* **2024**, *55*, 105323. https://doi.org/10.1016/j.surfin.2024.105323.
(74) Sharma, N.; Sharma, S. Analyzing the Effect of Chirality and Defects on Mechanical Properties of Carbon Nanotube Reinforced Polycarbonate Composites Using Molecular Dynamics. *Model. Simul. Mater. Sci. Eng.* **2022**, *30* (4), 045011. https://doi.org/10.1088/1361-651X/ac64b7.
(75) Yang, K.; Wang, S.; Chen, Y.; Dong, H.; Wang, Q.; Cheng, Q. Dynamic Mechanical Properties and Energy Absorption Capabilities of Polyureas Through Experiments and Molecular Dynamic Simulation. *Polymers* **2025**, *17* (1), 107. https://doi.org/10.3390/polym17010107.
(76) Michaels, W.; Zhao, Y.; Qin, J. Atomistic Modeling of PEDOT:PSS Complexes II: Force Field Parameterization. *Macromolecules* **2021**, *54* (12), 5354–5365. https://doi.org/10.1021/acs.macromol.1c00860.
(77) Kıbrıs, E.; Barbak, N. N.; Irmak, N. E. CHARMM Force Field Generation for a Cationic Thiophene Oligomer with ffTK. *J. Mol. Model.* **2021**, *27* (2), 34. https://doi.org/10.1007/s00894-020-04610-2.
(78) Sundaram, V.; Lyulin, A. V.; Baumeier, B. Development and Testing of an All-Atom Force Field for Diketopyrrolopyrrole Polymers with Conjugated Substituents. *J. Phys. Chem. B* **2020**, *124* (48), 11030–11039. https://doi.org/10.1021/acs.jpcb.0c06787.
(79) Qiao, Y.; Luo, M.; Yin, Q.; Wang, Y.; Zhou, G. Molecular Dynamics Simulation Study on Thermal Transport in Graphyne/Polyaniline Composite System. *Macromol. Theory Simul.* **2023**, *32* (6), 2300023. https://doi.org/10.1002/mats.202300023.
(80) Li, J.; Jin, S.; Lan, G.; Chen, S.; Li, L. Molecular Dynamics Simulations on Miscibility, Glass Transition Temperature and Mechanical Properties of PMMA/DBP Binary System. *J. Mol. Graph. Model.* **2018**, *84*, 182–188. https://doi.org/10.1016/j.jmgm.2018.07.005.
(81) Zhang, Z.; Zhang, L.; Jasa, J.; Negahban, M.; Gazonas, G. Molecular Sources of Ratcheting in Poly-Dispersed Polycarbonate. *Int. J. Fatigue* **2022**, *154*, 106567. https://doi.org/10.1016/j.ijfatigue.2021.106567.
(82) Skountzos, E. N.; Mermigkis, P. G.; Mavrantzas, V. G. Molecular Dynamics Study of an Atactic Poly(Methyl Methacrylate)–Carbon Nanotube Nanocomposite. *J. Phys. Chem. B* **2018**, *122* (38), 9007–9021. https://doi.org/10.1021/acs.jpcb.8b06631.
